# Carbonic anhydrase IX-targeted H-APBC nanosystem combined with phototherapy facilitates the efficacy of PI3K/mTOR inhibitor and resists HIF-1α-dependent tumor hypoxia adaptation

**DOI:** 10.1186/s12951-022-01394-w

**Published:** 2022-04-12

**Authors:** Jie Liu, Xiaochun Hu, Lei Feng, Yun Lin, Shujing Liang, Zhounan Zhu, Shuo Shi, Chunyan Dong

**Affiliations:** Breast Cancer Center, Shanghai East Hospital, Shanghai Key Laboratory of Chemical Assessment and Sustainability, School of Chemical Science and Engineering, Tongji University, Shanghai, 200092 People’s Republic of China

**Keywords:** Hypoxia, CAIX, Intracellular acidification, mTOR signal, Hypoxia adaptation

## Abstract

**Background:**

Non-redundant properties such as hypoxia and acidosis promote tumor metabolic adaptation and limit anti-cancer therapies. The key to the adaptation of tumor cells to hypoxia is the transcriptional and stable expression of hypoxia-inducible factor-1 alpha (HIF-1α). The phosphorylation-activated tumorigenic signal PI3K/AKT/mTOR advances the production of downstream HIF-1α to adapt to tumor hypoxia. Studies have elucidated that acid favors inhibition of mTOR signal. Nonetheless, carbonic anhydrase IX (CAIX), overexpressed on membranes of hypoxia tumor cells with pH-regulatory effects, attenuates intracellular acidity, which is unfavorable for mTOR inhibition. Herein, a drug delivery nanoplatform equipped with dual PI3K/mTOR inhibitor Dactolisib (NVP-BEZ235, BEZ235) and CAIX inhibitor 4‐(2‐aminoethyl) benzene sulfonamide (ABS) was designed to mitigate hypoxic adaptation and improve breast cancer treatment.

**Results:**

ABS and PEG-NH_2_ were successfully modified on the surface of hollow polydopamine (HPDA), while BEZ235 and Chlorin e6 (Ce6) were effectively loaded with the interior of HPDA to form HPDA-ABS/PEG-BEZ235/Ce6 (H-APBC) nanoparticles. The release of BEZ235 from H-APBC in acid microenvironment could mitigate PI3K/mTOR signal and resist HIF-1α-dependent tumor hypoxia adaptation. More importantly, ABS modified on the surface of H-APBC could augment intracellular acids and enhances the mTOR inhibition. The nanoplatform combined with phototherapy inhibited orthotopic breast cancer growth while reducing spontaneous lung metastasis, angiogenesis, based on altering the microenvironment adapted to hypoxia and extracellular acidosis.

**Conclusion:**

Taken together, compared with free BEZ235 and ABS, the nanoplatform exhibited remarkable anti-tumor efficiency, reduced hypoxia adaptation, mitigated off-tumor toxicity of BEZ235 and solved the limited bioavailability of BEZ235 caused by weak solubility.

**Graphical Abstract:**

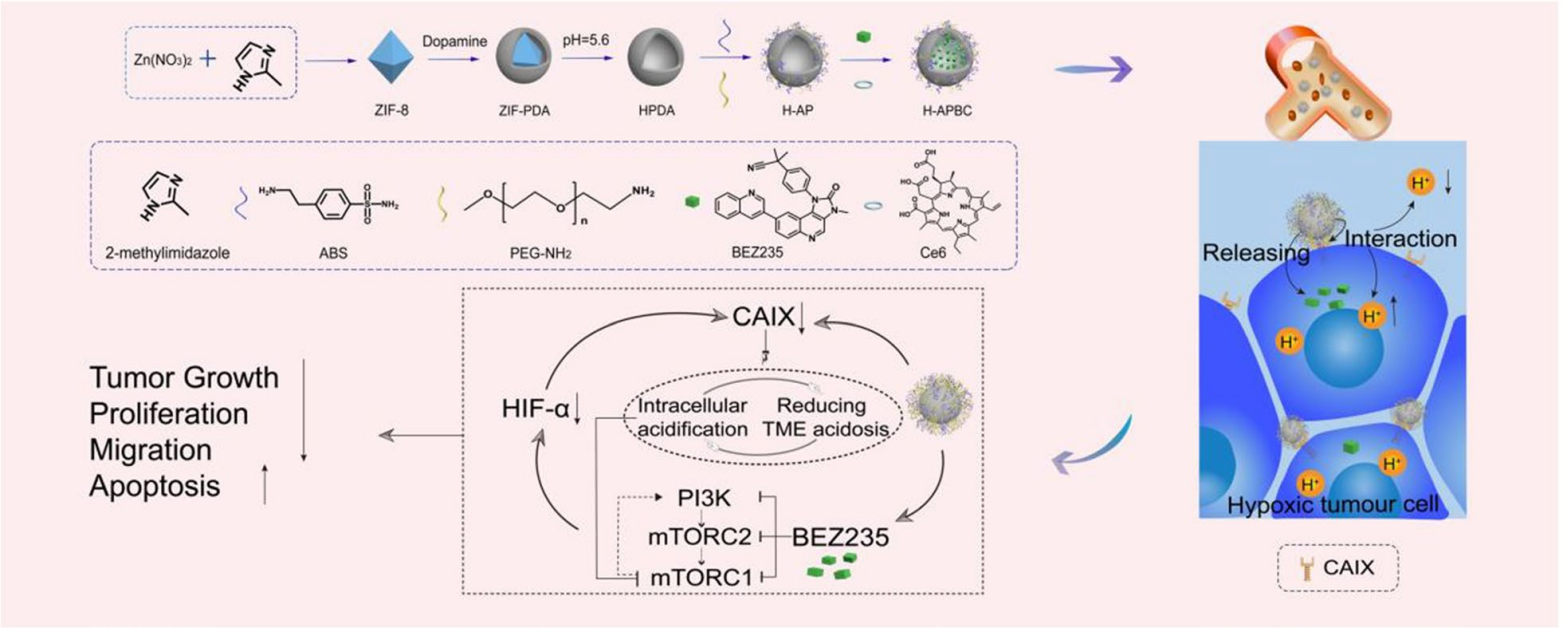

**Supplementary Information:**

The online version contains supplementary material available at 10.1186/s12951-022-01394-w.

## Introduction

As of 2020, breast cancer has replaced lung cancer to become the most commonly diagnosed malignancy around world, and to make matters worse, it has the highest mortality rate among female malignancies [1]. As a highly heterogeneous tumor, disordered metabolism is an obstacle to breast cancer treatment [2–4]. Oxygen is an essential substrate for metabolism in living organisms [5]. However, the disorganized structure or function of the tumor vascular network and the increased diffusion distances between rapidly proliferating tumor cells and blood vessels contribute to tumor hypoxia [6]. Under hypoxia, tumors produce multiple oncogenic transformations in response to activated hypoxia-inducible factor-1 alpha (HIF-1α) signal, which promote tumor adaptation to hypoxia [7, 8], such as metabolic adaptation, tumor microenvironment (TME) acidosis, distant metastasis and angiogenesis [9]. Furthermore, breast cancer patients expressing high levels of HIF-1α suffer from poorer disease-free survival (DFS) as well as worse overall survival (OS) [10]. Additionally, the tumor hypoxia regions usually coexist with TME acidosis [11, 12]. In normal tissues, intracellular pH (pHi) is typically ~ 7.2, which is a little lower compared to the extracellular pH (pHe) for ~ 7.4 on the basis of a well-circulated buffering system. However, cancer cells have a higher pHi of > 7.4 and a lower pHe of ~ 6.7–7.1 under hypoxia [13]. Carbonic anhydrase IX (CAIX), as the transmembrane protein with pH-regulatory effects, is a tumor-associated isoenzyme of carbonic anhydrases (CAs) family which is induced by HIF-1α under hypoxia and is rarely expressed in normal cells [14]. CAIX catalyzes the rapid hydration of extracellular CO_2_ to H^+^ and HCO_3_^−^, while HCO_3_^−^ returns to the cells via bicarbonate transporters, maintaining a tumor environment of extracellular acidic and intracellular alkaline [15]. Meanwhile, the reverse pH gradient inside and outside the tumor cells induces tumor to transform into an acid-resistant and pro-metastatic phenotype, which drives the malignant process by remodeling the extracellular matrix (ECM), promoting angiogenesis and immune escape [16–19].

Strategies to overcome tumor hypoxia adaptation include disrupting HIF signaling, suppressing TME acidosis, mediating metabolism and targeting hypoxic tumor cells pathways [20, 21]. As a key point to tumor anabolism, mTOR is mainly activated by phosphatidylinositol-3 kinase/protein kinase B/mammalian target of rapamycin (PI3K/AKT/mTOR) signal [22], which also regulates HIF-1α translation [23, 24]. Meanwhile, mTOR is a therapeutic target for tumors due to the function in promoting the synthesis of amino acids, glucose and other substances required for cancer cell growth, enabling tumors metastasis and anti-apoptosis [25]. Therefore, the inhibition of mTOR not only has a therapeutic effect but also reduces HIF-1α expression in breast cancer. mTOR includes two complexes, mTOR complex 1 (mTORC1) and mTOR complex 2 (mTORC2) [26]. However, the negative feedback activation for PI3K-AKT induced by mTORC1 suppression promotes tumor progression [27]. Dactolisib (NVP-BEZ235, BEZ235) is an inhibitor targeting PI3K and mTOR (mTORC1 and mTORC2), which avoids the feedback activation and produces a stronger inhibition for the positive feedback loop of PI3K/AKT/mTOR pathway [28]. Owing to the poor solubility, oral bioavailability of BEZ235 is weakened, which seriously limits the anti-tumor effects [29]. The study has elucidated that acid inhibits the translocation of mTORC1 from the cytoplasm to the perinuclear lysosomal surface, which prevents mTORC1 phosphorylation by PI3K/AKT [30]. As a tumor therapeutic agent, CAIX inhibitors can cause intracellular acidification [31]. 4‐(2‐aminoethyl) benzene sulfonamide (ABS) as a water-soluble CAIX inhibitor based on benzenesulfonamide can effectively inhibit CAIX, which has shown excellent anti-tumor effects in some research [32, 33].

Recently, with the emergence of ‘biomedical optics’ as a potential field with the benefits of non- or minimally invasive and highly selective, photodynamic therapy (PDT) and photothermal therapy (PTT) has become an emerging area of modern medicine. Studies have shown that the synergistic effect of PDT/PTT could enhance the effectiveness and reduce the limitations of each treatment modality [34, 35]. Photosensitizers (PSs)-mediated PDT generates reactive oxygen species (ROS) with toxic effects on tumor cells via the type I mechanism and the type II mechanism under laser irradiation [36–38]. Nonetheless, PDT relying on type II mechanism exacerbates tumor hypoxia and triggers angiogenesis via a signal cascade [39]. However, mTOR signaling activity is enhanced in perivascular cancer cells on account of the intense anabolism [40], suggesting the rationale for combining PDT with mTOR inhibitors. PTT can induce irreversible tissues damage by raising the tumor temperature above 42 °C to achieve thermal ablation [34, 35, 41, 42]. At the same time, the heat production of PTT can improve the blood flow rate in the tumor, facilitating the delivery of PSs or nanomaterials to the cells and enhancing the efficacy of PDT or nanomedicine [42, 43]. However, the pharmacokinetic properties of photosensitizers or PTT agents and drug molecules are usually different. Therefore, the development of suitable carriers to ensure the simultaneous therapeutic roles and tumor-specific accumulation in vivo is one of the best strategies for cancer treatment [44].

**Scheme 1:**
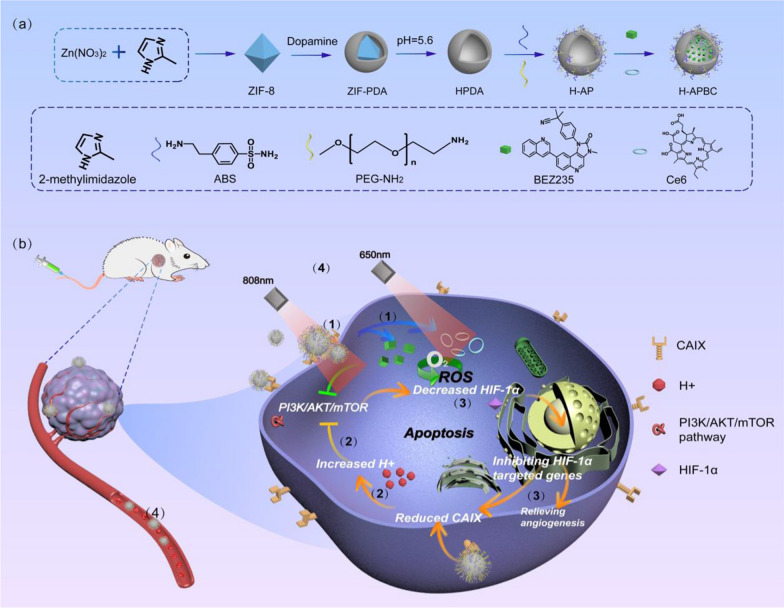
Schematic illustration of H-APBC synthesis and treatment. **a** Synthesis methods of the drug delivery nanoplatform H-APBC. **b** Schematic diagram of enhanced therapeutic effect via H-APBC in hypoxic tumor cells

For this research work, the drug delivery nanoplatform HPDA-ABS/PEG-BEZ235/Ce6 (H-APBC) in combination with phototherapy has been constructed to increase intracellular acidity, inhibit PI3K/mTOR signal and attenuate HIF-1α-dependent tumor hypoxia adaptation. As a naturally biopolymer formed by dopamine oxidation, polydopamine (PDA) exhibits superior biocompatibility and low cytotoxicity. Depending on π–π stacking and hydrogen bonding, PDA possess excellent drug-carrying capacity and acid responsive release properties. Meanwhile, PDA shows high photothermal conversion effectiveness depending on the wide range of absorption from ultraviolet (UV) to NIR wavelengths [45, 46]. Firstly, to facilitate the loading of drug molecules, hollow polydopamine (HPDA) nanoplatform with higher surface area and more internal space has been synthesized. Then, to optimize the biocompatibility for the nanoparticles (NPs) and to enable ABS delivery, HPDA-ABS/PEG (HAP) were constructed by modifying ABS and PEG-NH_2_ on the surface of HPDA. Finally, BEZ235 and photosensitizer Chlorin e6 (Ce6) were encapsulated within HAP to synthesize H-APBC nanocomposites (Scheme 1a). The anti-tumor effects of H-APBC were mainly reflected in (Scheme 1b): (1) Due to the enhanced permeability and retention (EPR) effect, H-APBC accumulated in tumor tissues. The modified ABS on the surface of H-APBC could recognize transmembrane protein CAIX and bound to the cell membrane. Then H-APBC NPs were adsorbed on the cell membrane surface, which were in turn endocytosed by tumor cells. (2) The modified ABS on the surface of H-APBC attenuated the hydration of CO_2_ to H^+^ and HCO_3_^−^ in TME. The reduction of extracellular H^+^ decreased the acidity of TME. At the same time, the HCO_3_^−^ entering the cells via the bicarbonate transporters was reduced, which in turn inhibited intracellular alkalinization and increased intracellular accumulation of H^+^. This facilitated the mitigation of cancer cell migration and improved the inhibition of mTORC1. (3) H-APBC presented acid-responsive release performance of Ce6 and BEZ235. The released BEZ235 and intracellular acids inhibited the PI3K/AKT/mTOR pathway and suppressed HIF-1α expression which decreased the downstream CAIX production and angiogenesis. (4) H-APBC combined Ce6-mediated PDT and HPDA-mediated PTT to realize a multi-modal tumor killing strategy and reduce the recognized off-tumor toxicity of BEZ235 in systemic therapy.

## Experimental sections

### Materials

Zinc nitrate hexahydrate [Zn(NO_3_)_2_·6H_2_O, 98%], Dopamine hydrochloride (98%) and 2-methylimidazole (99%) were purchased from Aladdin (Shanghai, China). NVP-BEZ235 (CAS No. 915019-65-7) was purchased from MedChemExpress Europe (Sollentuna, Sweden). 4‐(2‐Aminoethyl) benzene sulfonamide (CAIX inhibitor) (CAS No. 35303-76-5) was purchased from MACKLIN (Shanghai, China). Cell counting kit-8 (CCK-8) and 4, 6-diamidino-2-phenylindole (DAPI) were obtained from KeyGEN BioTECH (Shanghai, China). Paraformaldehyde (4%) was issued by DingGuo Biotech. Co., Ltd. (Shanghai, China). High glucose Dulbecco’s modified Eagle’s medium (DMEM), 1% penicillin–streptomycin (PS), 0.25% trypsin–EDTA and phosphate buffered saline (PBS) were purchased from Hyclone (Logan, Utah, USA). Fetal bovine serum (FBS) was acquired from Gibco Invitrogen (USA). The transwell chamber (3422) was obtained from Corning (Corning, NY, USA). Reactive oxygen species assay kit based on DCFH-DA (2,7-dichlorodihydrofluorescein diacetate) and pH fluorescent probe (BCECF-AM) were purchased from Beyotime Biotechnology (Shanghai, China).

### Characterization

Transmission electron microscopic (TEM) was carried out on a TEOL JEM-2100 transmission electron microscope. Scanning electron microscopy (SEM) results were acquired with a Hitachi S-4800 scanning electron microscope. Powder X-ray diffraction was obtained by a Bruker D8 VENTURE X-ray diffractometer with Cu Ka radiation source. The PTT study was carried out with an 808 nm NIR laser (Changchun Laser). The photothermal conversion was monitored with a thermal infrared imager (DALI TECHNOLOGY).

### Synthesis of HPDA

ZIF-8 and ZIF-PDA were prepared by the reported method [47]. ZIF-PDA (30 mg) was dispersed in PBS (15 mL, 5 mM, pH = 5.6), and the mixture was kept in an oil-bath and stirred for 10 min under 50 ℃. The hollow product was centrifuged at 10,000 rpm for 10 min, as well as several washes thoroughly with water.

### Synthesis of HPDA-ABS/PEG (H-AP)

ABS (20 mg) and PEG-NH_2_ (10 mg) were dissolved in Tris-buffer (10 mL, 10 mM, pH = 8.5). Five minutes later, HPDA (20 mg) was added to the above solution with stirring through the night. The product was obtained by centrifugation (10,000 rpm, 10 min), as well as several washes with water to remove uncombined ABS and PEG-NH_2_.

### Synthesis of HPDA-ABS/PEG-BEZ235/Ce6 (H-APBC)

BEZ235 (5 mg) and Ce6 (2.5 mg) were dissolved in methanol (5 mL), and then, H-PEG/ABS (10 mg) was added into the above mixture and stirred during the night at room temperature. The product was collected by centrifugation at 10,000 rpm for 10 min and washed with water for several times to remove uncombined BEZ235 and Ce6. Finally, the loading capacity (LC) of BEZ235 and Ce6 were calculated as follows formulae, respectively:

$$\begin{aligned}{\text{LC wt.}}\%{\text{ of BEZ235}}\, & = \,\left( {{\text{M}}_{{{\text{BEZ235}}}} - {\text{M}}_{{\text{BEZ235 - supernanant}}} } \right)\\ &\quad/{\text{M}}_{{{\text{H}} - {\text{APBC}}}} \times 100\%\end{aligned}$$

$${\text{LC wt}}{.}\% {\text{ of Ce6}}\, = \,\left( {{\text{M}}_{{{\text{Ce6}}}} - {\text{M}}_{{{\text{Ce6}} - {\text{supernanant}}}} } \right)/{\text{M}}_{{{\text{H}} - {\text{APBC}}}} \times 100\%$$

The loading capacity of Ce6 and BEZ235 was about 12.5 wt.% and 17.4 wt.%, respectively. Furthermore, the ABS loading capacity was calculated to be 24.2 wt.% determined by the sulfur element ration in H-APBC based on the inductively coupled plasma optical emission spectrometry (ICP-OES) test.

HPDA-PEG-BZE235/Ce6 (H-PBC) and HPDA-ABS/PEG-BEZ235 (H-APB) were obtained by same method.

### In vitro photothermal performance

The photothermal conversion efficiency of H-APBC was evaluated by the temperature variation of H-APBC suspensions with different concentrations (0–400 ppm) or different power intensity of 808 nm laser. The temperature variations were monitored and imaged simultaneously with a thermal infrared imager.

### In vitro singlet oxygen (^1^O_2_) generation

Dichlorodihydrofluorescein (DCFH) was employed as an indicator for the detection of ^1^O_2_. Briefly, DCFH solution (10 μL, 2 mM) was mixed with H-APBC dispersion (1 mL, 10 μg mL^−1^). Then the mixture was treated with irradiation at 650 nm (50 mW cm^−2^), and increasing DCFH fluorescence was measured by the FL spectrophotometer. And the generation of ^1^O_2_ was related to the increasing fluorescence intensity of DCFH linearly. The experiment of the control group H-APB was conducted with the same steps.

### Cell culture

4T1 mouse breast cancer cells were obtained from ATCC, and the ATCC number is ATCC^®^ CRL-2539TM. The 4T1 cells were cultured in DMEM doped with 10% FBS and 1% PS in a humid incubator at 37 °C with 5% CO_2_. During the hypoxia experiment, 4T1 cells were cultured at 37 °C in a hypoxic environment containing 1% O_2_ and 5% CO_2_.

### Cytotoxicity assay

Approximately 1 × 10^4^ cells per well were seeded in 96-well plates with complete medium (100 μL) and cultured under normoxic/hypoxic conditions. The cells were then exposed to different concentrations of HPDA and H-APBC. After 12 h, the medium was replaced with DMEM (without 10%FBS) containing 10% CCK-8 reagent. After 2 h of incubation, cell viability was calculated by measuring the absorbance at 450 nm with the exclusion at 630 nm.

### Cellular uptake of H-APBC

Flow cytometry (CytoFLEX LX, USA) and CLSM (Leica SP8) were used to verify cellular uptake. 1 × 10^5^ and 2 × 10^5^ 4T1 cells per well were cultured in 12-well plates and CLSM-exclusive culture dishes in hypoxic environment, respectively. Then, these 4T1 cells were exposed to H-APBC (50 μg mL^−1^) for 0–6 h, after which the cells were quantified by flow cytometry and observed under CLSM. Filter: Ce6 (excitation 405 nm; emission 650 nm).

### Intracellular H^+^ accumulation

The regulation of H-APBC on cellular acidity was measured using the intracellular pH indicator fluorescent probe BCECF‐AM (final concentration of 5 µmol L^−1^) and detected with CLSM and flow cytometry. 4T1 cells in CLSM-specific culture dishes were treated with H-APBC (50 μg mL^−1^) for 12 h and then stained with BCECF-AM for 30 min in normoxic or hypoxic environment. CLSM was used to observe the fluorescence images. 4T1 in 12-well plates were exposed to H-APBC (50 μg mL^−1^) for 12 h under hypoxia. The other 4T1 cells in 12-well plates cultured under hypoxic condition were treated with H-PBC (50 μg mL^−1^) for 12 h. These cells were prepared as cell suspension and stained with BCECF-AM for 30 min prior to the flow cytometry experiments. Filter: BCECF-AM (excitation 488 nm; emission 535 nm).

### Analysis of cell migration in vitro

For the migration assay, 4T1 cells were subjected to hypoxia or normoxia conditions for 6 h with different groups of drugs. 5 × 10^4^ cells were then collected and inoculated into the upper chamber (pore size 8 μm) with FBS-free medium, and the lower chamber was filled with DMEM medium (600 μL) containing 10% FBS. After incubation for 12 h, the migrating cells on the bottom side of the basal membrane were fixed with 4% formaldehyde and stained with 1% crystal violet, while non-migrating cells were removed. Cells were observed under a light microscope.

### Intracellular ROS generation of H-APBC

The probe DCFH‐DA was utilized to examine ROS production under 650 nm laser irradiation (50 mW cm^−2^, 2 min) in a hypoxic condition. CLSM-exclusive culture dishes were plated with 2 × 10^5^ 4T1 cells per well under hypoxia. Afterwards, 4T1 cells were exposed to varying concentrations of H-APBC (0, 25, 50 and 100 μg mL^−1^) for 12 h during the laser irradiation, and the images of the cells were taken by CLSM.

### Cell apoptosis assay

For 4T1 cell apoptosis, cell apoptosis detection kit was selected and then analyzed by flow cytometry. Firstly, 4T1 cells were seeded in 12-well plates and treated with H-APBC (50 µg mL^−1^) for 12 h under the hypoxic/normoxic atmosphere. Next, these cells were given PDT (650 nm laser, 50 mW cm^−2^, 2 min), PTT (808 nm laser, 2 W cm^−2^, 2 min) and PDT (650 nm laser, 50 mW cm^−2^, 2 min) combined with PTT (808 nm laser, 2 W cm^−2^, 2 min) treatment. Finally, cells were collected and stained for further cytometric analysis.

### In vitro synergetic anti-tumor efficiency

The killing efficacy of different treatments on 4T1 cells was analyzed using the CCK-8 method mentioned above. 4T1 cells cultured in 96-well plate using complete medium (100 µL) in hypoxic environment were incubated with another FBS-free medium (100 µL) which contain different concentrations of drugs (0, 25, 50, 75 and 100 µg mL^−1^) for 12 h. Then, the laser irradiation with PDT, PTT or combined PDT and PTT were performed (PDT: 650 nm, 50 mW cm^−2^, 2 min and PTT: 808 nm, 2 W cm^−2^, 2 min). Cells without PDT/PTT or with free BEZ235/ABS treatment were served as controls.

### Ethics statement

All experiments involving animals were in accordance with the Guide for the Care and Use of Laboratory Animals and carried out according to the ethical policies and procedures approved by the ethics committee of School of Medicine, of Tongji university (Approval No. TJBB00721103).

### Animal models establishing

Four to 5-week-old BALB/c mice (female) obtained from Shanghai Experimental Animal Center (Shanghai, China) and raised in the SPF laboratory of Tongji University were used to explore antitumor effects of H-APBC. 1 × 10^6^ 4T1 cells were orthotopically implanted into the fat pad of left mammary of mice. The tumor size of each mouse was corded during treatment to calculate the tumor volume and evaluate the anti-tumor efficacy in different treatment groups. Mouse weight was also recorded at the same time. Mice were executed after treatment in each group, and the organs, tumors and serum were taken to ensure subsequent analysis.

### In vivo photothermal and fluorescence imaging

For photothermal imaging, each mouse was injected with H-APBC (100 μL, 14 mg kg^−1^, an equivalent dose of 2.5 mg BEZ235 per kg) solution through the tail vein when tumor volume was up to about 200 mm^3^, and the control group was injected with PBS (100 μL) in the same way. The temperature changes at the tumor site during PTT in mice were recorded using LTX3-P Infrared Imaging Technique. For fluorescence imaging, similar mice (tumor volume = 200 mm^3^) in the experimental group and control group were also injected with H-APBC (100 μL, 14 mg kg^−1^, an equivalent dose of 2.5 mg BEZ235 per kg) solution and PBS (100 μL) through the tail vein, respectively. Then mice were anesthetized and imaged using a small animal imaging system (Aniview; excitation wavelength 405 nm and emission wavelength 650 nm) at given time points (0, 1, 3, 6, 12, and 24 h after injection). Finally, mice were euthanized after 24 h injection, and then tumors and critical organs were obtained for ex vivo imaging analysis.

### In vivo anti-tumor efficacy evaluation

The 4T1 breast cancer mice models were randomly divided into 7groups (n = 5, the administration volume of drugs was 100 μL): (1) PBS, (2) ABS (3) BEZ235 (4) H-APBC (5) H-APBC with laser (650 nm, 50 m W cm^−2^, 5 min) (6) H-APBC with laser (808 nm, 2 W cm^−2^, 5 min) (7) H-APBC with laser (650 nm, 50 mW cm^−2^, 5 min and 808 nm, 2 W cm^−2^, 5 min). The concentrations of ABS, BEZ235, H-APBC were 3.5 mg kg^−1^ (based on the loading rate of ABS in H-APBC), 2.5 mg kg^−1^ (based on the loading rate of ABS in H-APBC) and 14 mg kg^−1^ (an equivalent dose of 2.5 mg BEZ235 per kg). The group treatment was started when the tumor sizes were up to approximately 100 mm^3^. Length (L) and width (W) of the tumors and mice body weight were recorded to calculate tumor size (V) (V = L × W^2^/2). After 14th day’s treatment, all mice were euthanized, and tumors and major organs (heart, liver, spleen, lung and kidney) were excised for further experiments. Tumor tissues were used for subsequent hematoxylin and eosin (H&E) analysis, ROS generation stained by dihydroethidium (DHE), immunohistochemistry, immunofluorescence and terminal-deoxynucleotidyl transferase mediated nick end labeling (TUNEL) experiments. Main organs were used for H&E analysis.

### Blood biochemical assay

One point five mL of blood were taken from each mouse and placed in SST tubes at room temperature for 2 h, then centrifuged at 3000 rpm, 4 °C for 15 min, and the supernatant serum was analyzed for hepatic and renal function.

### Statistical analysis

All experiments were repeated independently at least three times. One-way analysis of variance (ANOVA) was performed to compare the differences between groups. Data are expressed as mean ± standard deviation. Statistical significance of the difference was expressed as p value * < 0.05, ** < 0.01, *** < 0.001.

## Results and discussion

### Preparation and characterization of H-APBC

**Fig. 1:**
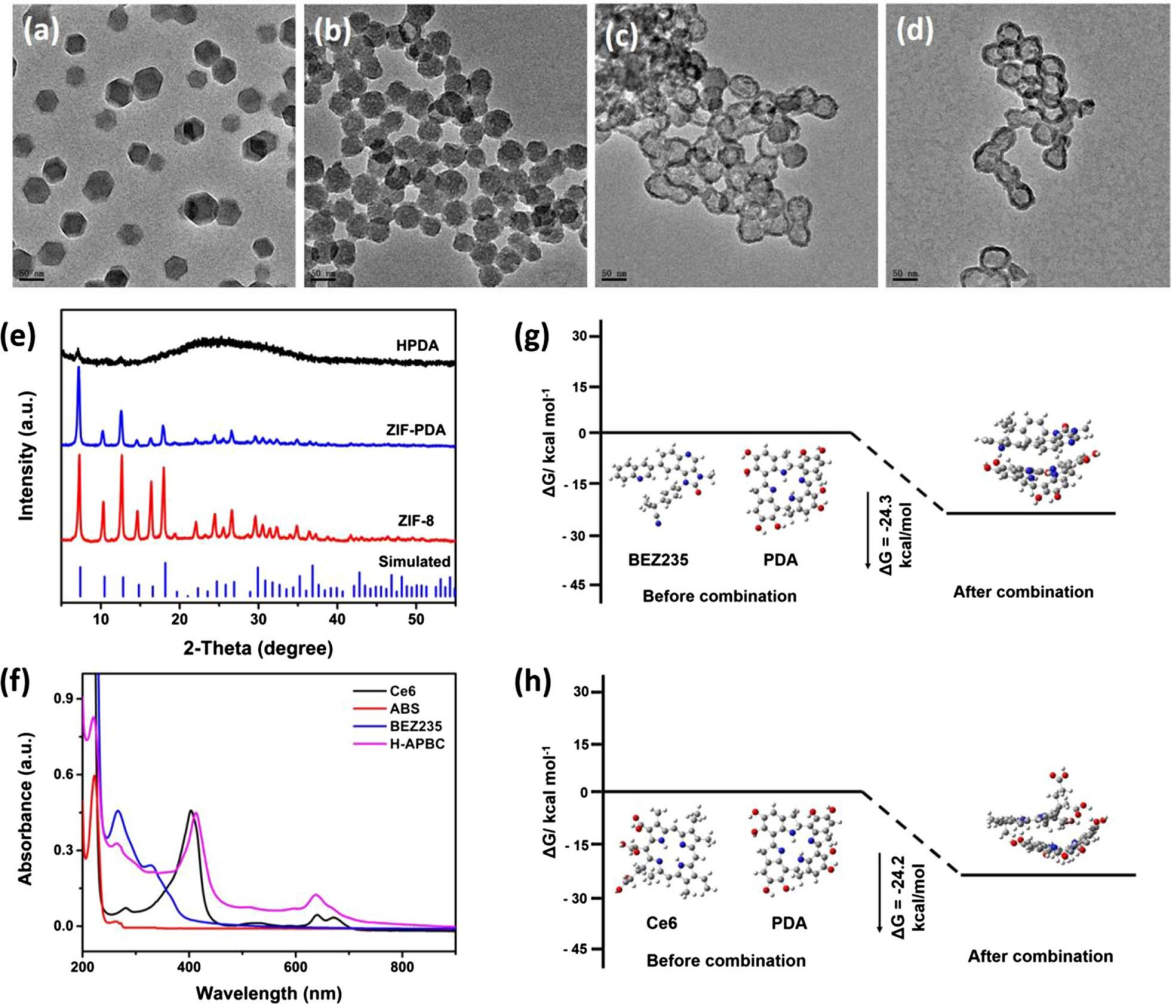
Preparation and characterization of H-APBC. TEM images of **a** ZIF-8, **b** ZIF-PDA, **c** HPDA, **d** H-APBC. **e** XRD patterns of ZIF-8, ZIF-PDA and HPDA. **f** UV–vis–NIR absorption spectra of H-APBC and free components. The energy variation of BEZ235 (**g**) or Ce6 (**h**) and PDA before combination and after combination. The DFT calculation was carried out by Gaussian 09 with b3lyp/6-31 g* (PCM) method

First of all, zeolitic imidazolate framework 8 (ZIF-8) was built by zinc nitrate hexahydrate and 2-methylimidazole (Fig. 1a and Additional file 1: Fig. S1). Then, the polydopamine layer was generated under alkaline condition, and the ZIF-PDA nanoparticles with core–shell structure were obtained (Fig. 1b). Next, the core was removed on account of the acid lability property of ZIF-8, and the TEM image (Fig. 1c) indicated the ‘core’ completely disappeared and HPDA formed. In addition, the structure of the product was verified by the X-ray diffraction (XRD). The diffraction patterns of ZIF-PDA agreed with that of pure ZIF-8, while for HPDA, the diffractions peaks of ZIF-8 almost disappeared, which was a further proof of the formation of hollow dopamine (Fig. 1e). ABS and PEG-NH_2_ was attached on the surface of polydopamine by Michael addition reaction [48]. Finally, BEZ235 and Ce6 were co-loaded on HPDA to the final product (H-APBC) (Fig. 1d). In UV–vis absorption spectra of H-APBC (Fig. 1f), typical absorption peaks at 220 nm, 265 nm and 412 nm were observed for ABS, BEZ235 and Ce6, respectively, demonstrating the successful loading of ABS, BEZ235 and Ce6. The theoretical calculation data revealed that the binding energy of BEZ235 and Ce6 with HPDA was − 24.3 kcal mol^−1^ and − 24.2 kcal mol^−1^, respectively (Fig. 1g, h), intermolecular force, hydrogen bonding and π–π stacking might favor the binding between BEZ235/Ce6 and HPDA.

To study the acid-responsive release performance of the Ce6 and BEZ235, H-APBC was incubated in solutions at different pH values to simulate the physiological environment in normal cells and tumor. Compared with pH 7.4, more Ce6 and BEZ235 were released in acid solution (Additional file 1: Fig. S2). Approximately 45.1% loaded Ce6 and 30.2% loaded BEZ235 were released from H-APBC after incubation under acid condition (pH 5.6) after 24 h incubation, which was much higher than those at pH 7.4. That could be attributed to the depolymerization of PDA layer in the acidic environment [49]. Therefore, the acid-responsive H-APBC could be applied to release drugs in acid tumor-microenvironment to induce multi-therapy.

### Photodynamic and photothermal effects measurement

**Fig. 2:**
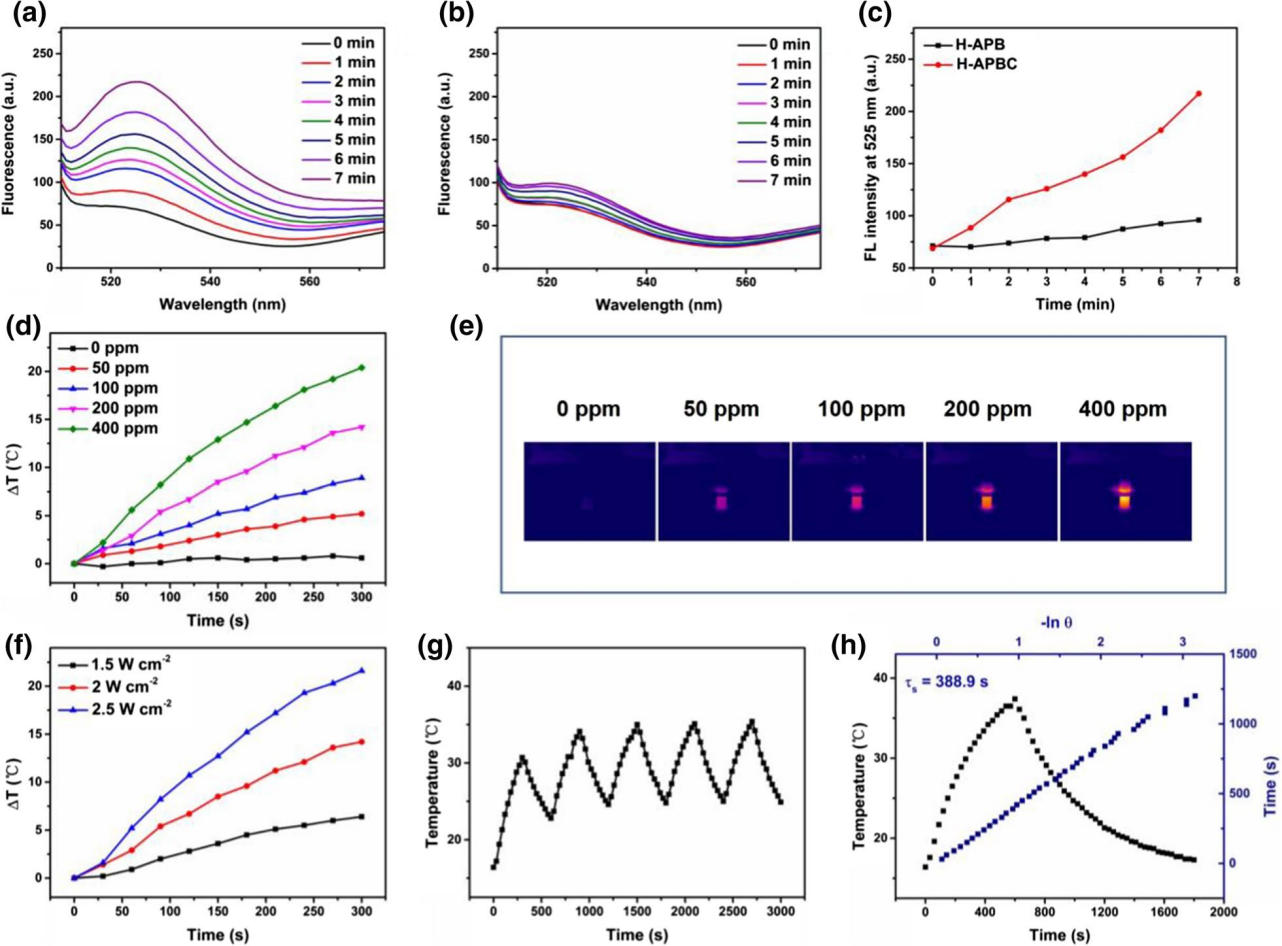
The photodynamic and photothermal effects of H-APBC. Time-dependent emission spectra of DCFH upon 650 nm laser irradiation for H-APBC (**a**) and H-APB (**b**). **c** Comparison of the average increase rate of DCFH intensity at 525 nm in the presence of H-APB or H-APBC. Temperature elevation (**d**) and the infrared temperature (IRT) images (**e**) of H-APBC with different concentrations under 808 nm laser irradiation for 300 s. **f** The photothermal heating curves of H-APBC under various irradiation energies (200 ppm). **g** Temperature variation of H-APBC (200 ppm) with 808 nm laser (2 W cm^−2^) switched on and off for five cycles. **h** Plot of cooling time vs. − lnθ and the photothermal effect of an aqueous dispersion of H-APBC under 808 nm laser irradiation for 300 s

The time-dependent fluorescence emission of DCFH was monitored to evaluate the ROS generation of H-APBC under 650 nm laser irradiation. The fluorescence intensity increased obviously in the presence of H-APBC, which was attributed to the outstanding photocatalytic activity of H-APBC and the continuous ROS production during 650 nm laser irradiation (Fig. 2a). In contrast, for H-APB group, which was not modified with photosensitizer Ce6, there was no distinct changes of fluorescence intensity under the same experiment condition (Fig. 2b, c). The absorption intensity of H-APBC dispersion at 808 nm increased linearly with the increasing concentration of H-APBC (Additional file 1: Fig. S3), which revealed H-APBC presented the outstanding solubility. Then, the photothermal performances were evaluated under 808 nm laser irradiation. As depicted in Fig. 2d–f, the temperature changes of H-APBC with gradient concentrations (0–400 ppm) and the temperature variation of H-APBC under different laser power (1.5–2.5 W cm^−2^) possessed the concentration and laser power dependence. Among them, the temperature of the H-APBC dispersion (100 ppm) could increase 9.5 ℃, however, the temperature of the control group (pure water) just changed from 17.1 to 17.7 ℃. Moreover, after 5 cycles irradiation, the photothermal conversion efficacy of H-APBC remained unchanged, indicating that H-APBC exhibited excellent photothermal stability (Fig. 2g). In addition, by Roper’s method [50], the photothermal conversion efficacy (η) of H-APBC was calculated to be 16.3% (Fig. 2h).

### In vitro cytotoxic properties and biological features of H-APBC

**Fig. 3:**
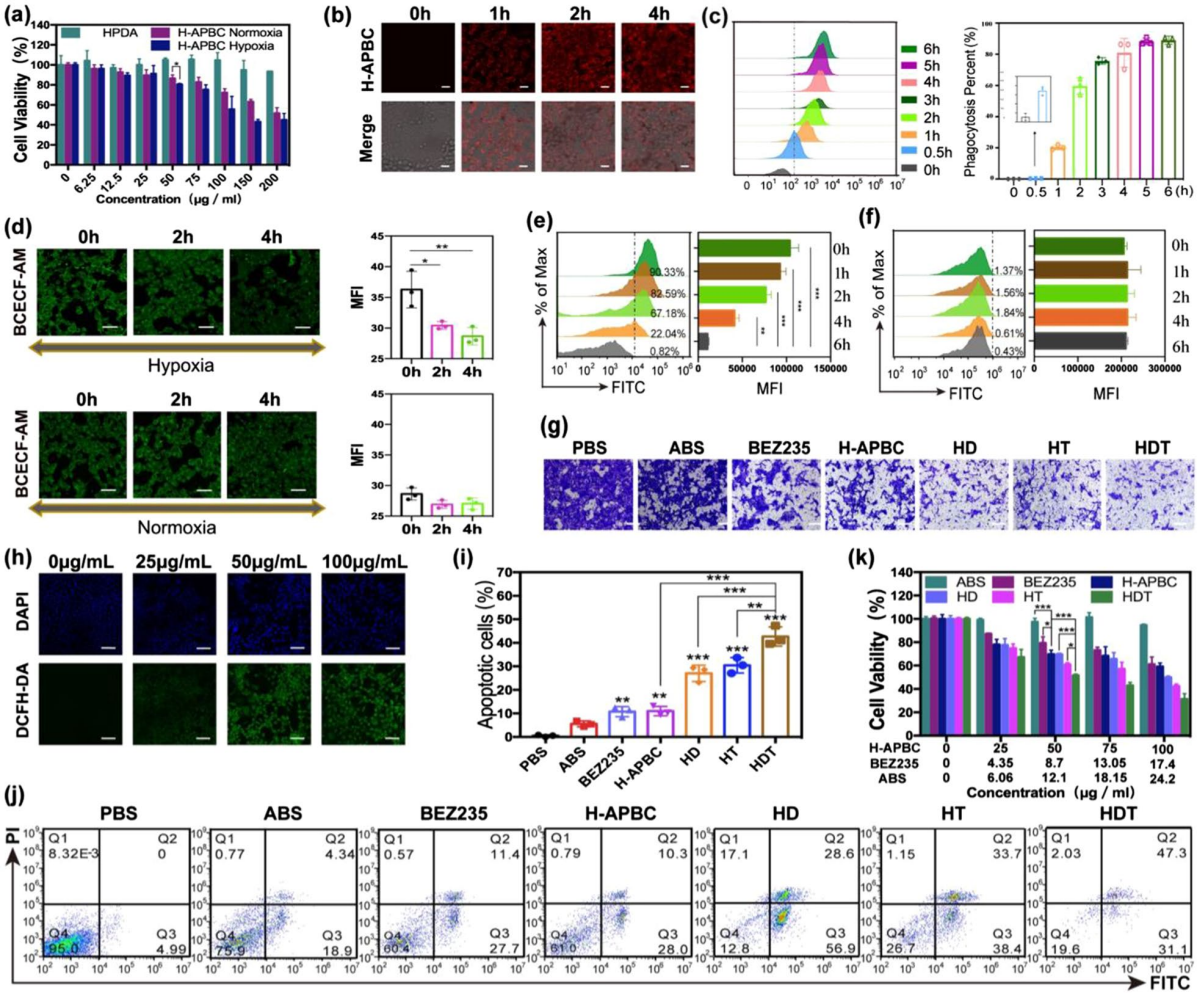
In vitro experiments with H-APBC. **a** Viability of 4T1 cells treated with HPDA and H-APBC without laser irradiation under the conditions of normoxic or hypoxic. (n = 3). CLSM images (**b**) and flow cytometric assay (**c**) [the average fluorescence intensity (left) and the percentage of cells taking up NPs (right)] of 4T1 cells treated with H-APBC at different time points under hypoxia. (n = 3). Scale bar: 25 μm. **d** CLSM images of intracellular acids detection in 4T1 cells with H-APBC under hypoxia or normoxia. Scale bar: 50 μm. Flow cytometry of intracellular acids in 4T1 cells treated with H-APBC (**e**) or ABS-free nanoparticles HPDA-PEG-BEZ235/Ce6 (**f**) under hypoxia. (n = 3). **g** Images of migrated 4T1 cells treated differently under hypoxia. Scale bar: 50 μm. **h** CLSM images of intracellular ROS detection for 4T1 cells treated with various concentrations of H-APBC with 650 nm laser under hypoxia. Scale bar: 75 μm. **i**, **j** Flow cytometry of the apoptotic 4T1 cells in different treatments during the hypoxia experiment. (n = 3) **k** Viability of 4T1 cells in different groups under the hypoxic condition. (n = 3). *p < 0.05, **p < 0.01, and ***p < 0.001

4T1 cells were selected to illustrate the effects of H-APBC for breast cancer bio-behavior. As shown in Fig. 3a, due to the interaction with CAIX and the release of BEZ235, H-APBC showed higher toxicity under hypoxia, which exhibited lower toxicity under normoxia as a result of the BEZ235 release only. Additionally, the cells viability was greater than 80% after 12 h of treatment, even when the maximum concentration of HPDA was 200 μg mL^−1^, reflecting the favorable biocompatibility of HPDA (Fig. 3a). According to the inhibition efficiency of H-APBC on 4T1 cells (Fig. 3a), 50 μg mL^−1^ was selected as the main cellular concentration with a treatment time of 12 h. The concentrations of corresponding pure drugs (BEZ235 and ABS) were calculated based on the loading rate in H-APBC (BEZ235: 8.7 μg mL^−1^, ABS: 12.1 μg mL^−1^). Then, based on the red fluorescence of Ce6, CLSM images demonstrated that H-APBC were strongly internalized by 4T1 cells (Fig. 3b and Additional file 1: Fig. S4). Flow cytometry analysis also confirmed that the average fluorescence intensity (Fig. 3c, left) and the percentage of cells taking up NPs (Fig. 3b, right) were remarkably enhanced and increased, respectively.

In addition, the main role of CAIX is pH regulation, which makes TME acidic and keeps intracellular pH (pHi) around 7.4 [13]. H-APBC reversed the pH gradient inside and outside the hypoxia tumor cells by inhibiting CAIX. Firstly, due to the modified ABS on the surface of H-APBC, the NPs alleviated TME acidosis and increased intracellular acidity by inhibiting the hydration of CO_2_ to H^+^ in TME. Then, intracellular acids and the released BEZ235 would inhibit the PI3K/AKT/mTOR pathway and diminish the HIF-1α expression in tumor cells. Finally, the lower HIF-1α attenuated the production of downstream CAIX. Intracellular acidification was analyzed by CLSM and flow cytometry, which were quantified with mean fluorescence intensity (MFI). Both CLSM and flow cytometry analysis showed a significant augment in intracellular acids (diminished green fluorescence density labeled by BCECF-AM) under hypoxia treated with H-APBC (Fig. 3d, upper part and Fig. 3e). Under normoxia, the green fluorescence density diminished insignificantly due to the lack of CAIX (Fig. 3d, lower part). Likewise, as a control, ABS-free nanoparticles HPDA-PEG-BEZ235/Ce6 displayed no effect on acid–base regulation, which lacked the ability to inhibit CAIX under hypoxia (Fig. 3f). Moreover, the acidic TME decreases intercellular adhesion and causes ECM lesions or breakdown, which accelerates cells metastasis [51, 52]. The migrating cells evidenced that the H-APBC group, especially the H-APBC + PDT (abbreviated as HD), H-APBC + PTT (abbreviated as HT) and H-APBC + PDT + PTT (abbreviated as HDT) treatment groups inhibited tumor migration more efficiently under hypoxia (Fig. 3g and Additional file 1: Fig. S5).

Studies have identified that mTOR-dependent augmented level of anabolism generates in cells adjacent to tumor capillaries [40]. HIF-1α-induced tumor angiogenesis is promoted by hypoxia, and PDT exacerbates this process. Therefore, the enhanced angiogenesis accompanied by the increased mTOR activity improved the sensitivity of H-APBC to tumors. ROS production (DCFH-DA-labeled green fluorescence) could be identified and the fluorescence signals were stronger with higher H-APBC concentrations under 650 nm laser irradiation in a hypoxic environment (Fig. 3h). Furthermore, the anti-cancer effects of H-APBC were validated due to the blockade for CAIX, PI3K/mTOR signaling and the combination with phototherapy. Under normoxia, approximately 16.09% of 4T1 cells showed apoptosis after H-APBC treatment as a result of the released BEZ235 (Additional file 1: Fig. S6). Under hypoxia, as shown in Fig. 3i, j, H-APBC exhibited a greater capacity to induce apoptosis with an apoptosis rate reached 38.3%, which was caused by interaction with CAIX and the release of BEZ235, inducing intracellular acidosis and exerting enhanced mTORC1 inhibition. Furthermore, the groups treated with HD, HT or HDT exhibited the strongest late and early apoptosis rates, which were 28.6% and 56.9%, 33.7% and 38.4, 47.3% and 31.1%. Additionally, the cytotoxicity of different treatment groups was consistent with apoptosis under hypoxia, while the difference in cytotoxicity between H-APBC and BEZ235 groups was not statistically significant under normoxia (Fig. 3k and Additional file 1: Fig. S7).

### In vivo tumor accumulation and combined therapeutic efficacy of H-APBC

**Fig. 4:**
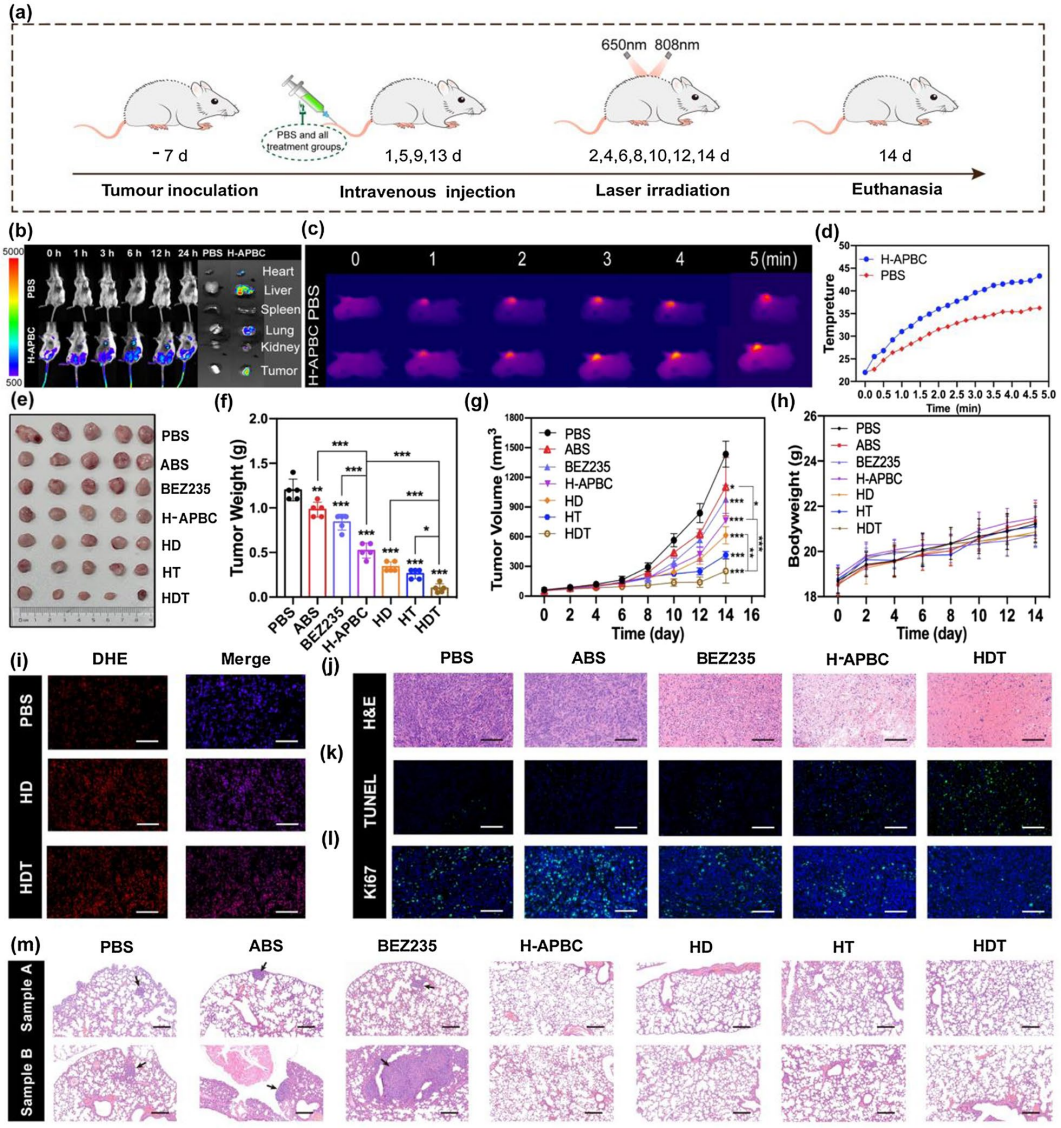
In vivo anti-tumor effects of H-APBC in animal model. **a** Treatment schedule. **b** Fluorescence images of mice after intravenous injection of H-APBC and ex vivo imaging of tumor and major organs after 24 h injection. **c** Photothermal effects of PBS and H-APBC under 808 nm laser irradiation. **d** Time-dependent manner of temperature change curve in tumor tissues. The representative photographs (**e**) and weight (**f**) of tumor tissues in different groups obtained on day 14 (n = 5). **g** Tumor growth and **h** body weight of mice in each group. **i** Fluorescence images of ROS production after different treatments. H&E (**j**), TUNEL (**k**), Ki67 (**l**) staining of tumor tissues in different groups (n = 5). Scale bar: 80 μm. **m** H&E staining of lung tissues in different groups (n = 5). Scale bar: 100 μm. *p < 0.05, **p < 0.01, and ***p < 0.001

Given the excellent application of H-APBC in vitro, the anti-cancer effects for H-APBC were further verified by the in vivo experiments (Fig. 4a). Firstly, the capacity of H-APBC to accumulate at the tumor sites was assessed by in vivo fluorescence imaging system. After H-APBC injection, the fluorescence intensity of tumor sites was progressively increased and reached peak during 6–12 h. Afterwards, local fluorescence in orthotopic tumor gradually diminished, indicating the clearance of H-APBC in mice. Twenty-four hours later, after euthanasia and excision of the mice, distinct fluorescence images were observed both in liver and tumor sites, suggesting that H-APBC accumulated rapidly in the tumor and was eventually metabolized by the liver (Fig. 4b). Moreover, the tumor in-situ thermography and real-time temperature were recorded at 12 h after injection of H-APBC using Infrared Imaging Technique under the laser irradiation. After 5 mins of irradiation, the local tumor temperature of the H-APBC group rapidly rose up to 43 °C and was sufficient to kill tumor cells, while a slight increase was observed in the PBS group (37 °C) (Fig. 4c, d).

After 14 days of treatment, the mice were euthanized. According to the volume and weight of tumors, the treatment groups revealed different anti-tumor effects compared to the PBS group (Fig. 4e–g). And all mice showed no remarkable difference in body weight (Fig. 4h). Particularly, the efficacy of H-APBC group was superior to that of free BEZ235 and ABS groups. On one hand, this was due to the preferential aggregation of H-APBC to tumors through the EPR effect, whereas BEZ235 and ABS groups were not predominantly distributed to tumor tissues. On the other hand, H-APBC enhanced intracellular acidification, exerted enhanced mTOR inhibition, and weakened HIF-1α-dependent hypoxia adaptation. Remarkably, the HDT group showed the strongest tumor growth inhibition. As an evidence for ROS production in tumors, DHE staining of tissue sections was performed and the noticeable red fluorescence was organized when treated with HD or HDT (Fig. 4i and Additional file 1: Fig. S8). H&E staining analysis of tumor tissues showed the most pronounced necrosis was in the HDT group, which could be clearly observed with karyorrhexis, karyolysis, intensely eosinophilic cytoplasm, and red-stained non-structural granular material formed by the fusion of necrotic cells and disintegrated intercellular matrix (Fig. 4j and Additional file 1: Fig. S9). These indicated that HDT was the most effective in promoting pathological damage, and similarly, TUNEL  staining and Ki-67  staining also showed that the HDT group had the strongest ability to induce apoptosis and inhibit proliferation in tumor tissues (Fig. 4k, l and Additional file 1: Fig. S9). Furthermore, solidly arranged irregular multilayered spontaneous lung metastatic cells from breast cancer with variable sized nucleus and distinct nuclear division were observed in the two mice lung tissue samples in the PBS, ABS and BEZ235 groups. Notably, no tumor-like lesions were found in the lung tissues in the H-APBC, HD, HT and HDT groups (Fig. 4m). All of these were the result of a combination of systemic treatment and local phototherapy with H-APBC, which demonstrated the apparent efficacy of the nanocomposite in breast cancer.

### Anti-hypoxia adaptation performance of H-APBC in vivo

**Fig. 5:**
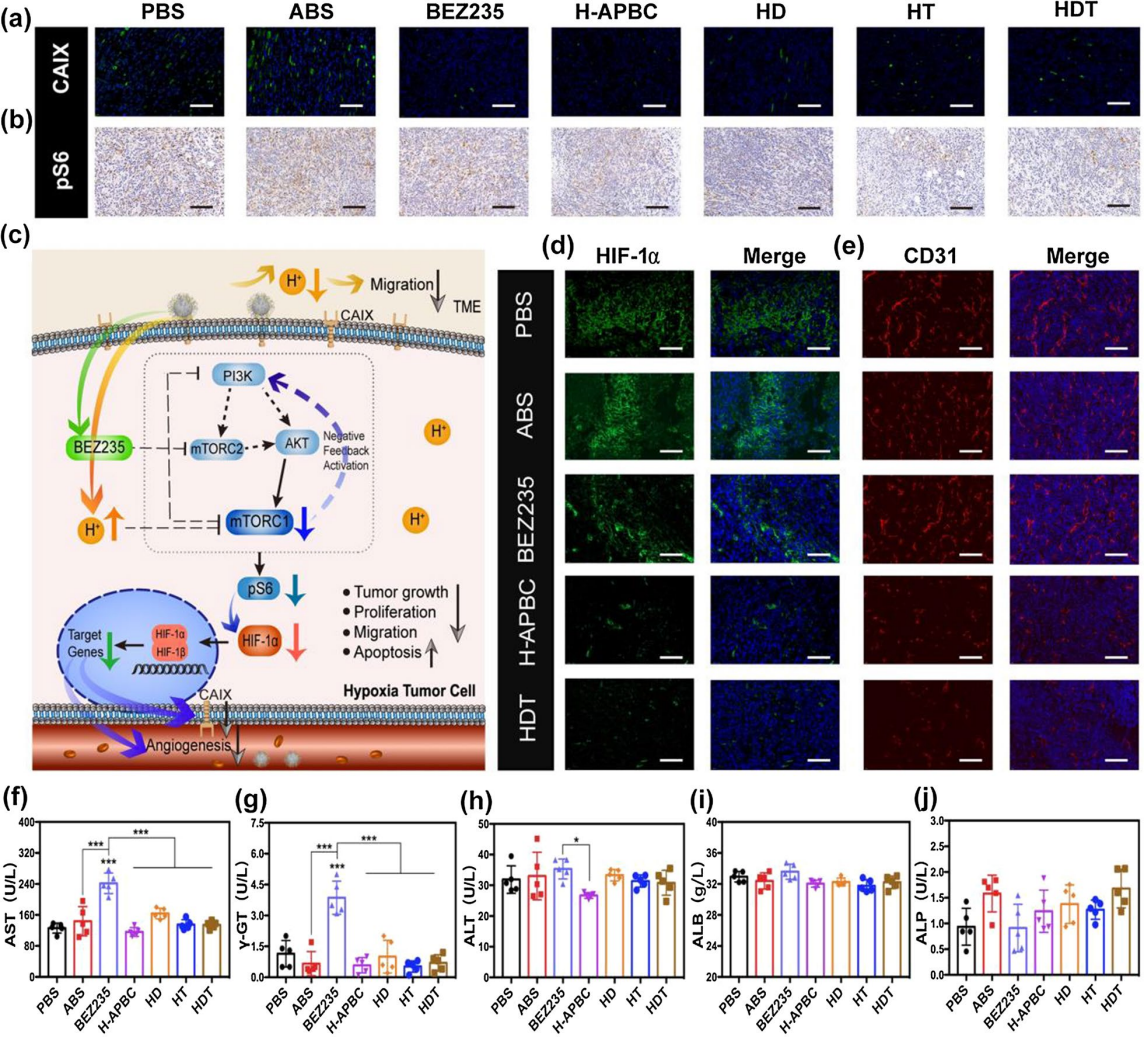
In vivo anti-hypoxia adaptation performance and biosafety assessment of H-APBC. Images of tumor tissues expressing CAIX (**a**) and pS6 (**b**) after different treatments. Scale bar: 80 μm (CAIX), 120 μm (pS6). **c** Anti-cancer strategy of H-APBC under hypoxic condition. Under hypoxia, H-APBC aggregated in tumor tissues, inhibited CAIX expression and released BEZ235 to attenuate TME acidosis, increase intracellular acidity, and exert the enhanced PI3K/AKT/mTOR pathway inhibition accompanied by decreased pS6 protein expression. H-APBC more strongly inhibited HIF-1α expression and mitigated metastasis and angiogenesis. Immunofluorescence images of expressed HIF-1α (**d**) and CD31 (**e**) in different groups. Scale bar: 80 μm (HIF-1α),120 μm (CD31). **f**–**j** Serum aspartate aminotransferase (AST), γ-glutamyltransferase (γ-GT), alanine aminotransferase (ALT), albumin (ALB) and alkaline phosphate (ALP) levels recorded for mice in the treatment groups. *p < 0.05, **p < 0.01, and ***p < 0.001

So far, H-APBC has exhibited outstanding performance towards tumor suppression through in vitro and in vivo research. Supported by its underlying mechanism, expression of the critical molecules in the tumor tissues was analyzed to adequately define the design concept. In this study, the ability to inhibit the expression of CAIX and PI3K/mTOR signal phosphorylation was the key point to exerting tumor inhibition by altering the tumor environment and regulating tumor anabolism. The expression of phosphorylated ribosomal S6 (pS6) protein downstream of PI3K/AKT/mTOR pathway was used to elucidate the inhibitory effect of H-APBC on this pathway. Histochemical analysis of tumor tissues confirmed that the CAIX and pS6 expression was attenuated in all H-APBC treatment groups with or without laser irradiation (Fig. 5a, b). As shown in Fig. 5c, PI3K/AKT/mTOR signal, as a major pro-survival pathway in cancer, also promoted the translation of HIF-1α [53–55], which regulated the expression of downstream CAIX. Therefore, besides the direct action of H-APBC on CAIX, H-APBC reduced a portion of CAIX production by inhibiting HIF-1α translation in hypoxic tumor tissues. For the reduction of pS6 expression, it was not only the result of H-APBC action on PI3K/AKT/mTOR pathway, but also the inhibitory effect of intracellular acids.

HIF-1α is a principal regulatory molecule for metabolic adaptation to hypoxia in solid tumors, inducing downstream target genes to convert tumor cells into a hypoxia-adapted phenotype with higher malignancy and heterogeneity [56, 57]. Angiogenesis is known to be strongly induced by hypoxia and TME acidosis [9, 39]. Immunofluorescence of tumor tissue sections showed that the HIF-1α expression was weakened, accompanied by decreased vascular density and severe vascular structure disruption (stained by the vascular endothelial cells marker CD31) in the H-APBC, HD, HT, HDT groups (Fig. 5d, e and Additional file 1: Fig. S10). All these proved the role of H-APBC in anti-tumor therapy and resistance against hypoxia adaptation.

### In vivo biosafety assessment after treatments

Furthermore, mTOR inhibitors feature pharmacological adverse event (AE) profile and the mTOR inhibitor-associated pneumonia with serious consequences is one of the AEs leading to treatment discontinuation [58]. So, mitigating the pharmacological AE of mTOR inhibitors is of great clinical importance. For long-term toxicity of the in vivo, hepatic and renal function, and the staining of key organs were selected as observation indicators. After the 14-day treatment period, biochemical analysis showed that the serum levels of serum aspartate aminotransferase (AST) and γ-glutamyltransferase (γ-GT) were evidently elevated within the free BEZ235 group. By comparison, hepatic or renal function were normal in the H-APBC, HD, HT, HDT groups, the NPs might suppress the release of BEZ235 into the circulation that lacked acid (Fig. 5f–j, Additional file 1: Fig. S11). Similarly, H&E staining also showed inflammatory cells infiltration in the liver portal areas and alveolar septum, with some alveolar cavities filled with edema fluid and red blood cells in the free BEZ235 group. Nevertheless, the organ inflammatory damage was significantly reduced in the H-APBC treated groups with or without laser irradiation, which still showed compensatory emphysema. These demonstrated that H-APBC significantly reduced the off-tumor toxicity of BEZ235. (Additional file 1: Fig. S12).

## Conclusion

Taking HPDA, which provided favorable biocompatibility and low cytotoxicity, as a carrier, H-APBC was developed for as an anti-cancer therapeutic agent in breast cancer, while also modifying the HIF-1-dependent tumor hypoxia adaptation. Under hypoxia, H-APBC was implemented as an anti-tumor strategy through reversal of the pH gradient inside and outside tumor cells, targeted therapy and the inhibition of tumor hypoxia adaptation (Fig. 5e). The microenvironment of hypoxia and acidosis alters original metabolic system of the tumor cells, converting them into phenotypes that are adapted to new metabolic pathways which weakens the medicaments therapeutic effects on different subgroups of patients [, 18, 19, 59]. Therefore, there is a compelling need to investigate treatments for TME. As the master switch for cellular anabolism, mTORC1 is critical for tumor cells growth and is a potent target for anti-tumor medicines. Although mTORC1 can be activated by multiple oncogenic pathways, the PI3K/AKT signal is the most common pathway for phosphorylation activation of mTORC1 in breast cancer [60]. H-APBC exerted greater antitumor efficiency by the following factors in hypoxic tumors. Firstly, H-APBC not only suppressed mTORC1 directly, but also augmented intracellular acidity by interaction with CAIX, which further enhanced the mTORC1 inhibition. Moreover, H-APBC could inhibit the positive feedback loop of PI3K/AKT/mTOR pathway by acting on PI3K and mTOR targets. Secondly, the restraint of mTORC1 facilitated the reduction of the downstream HIF-1α expression, which contributed to attenuate tumors resistance to hypoxia and mitigated HIF-1α-induced tumor angiogenesis. Then, due to the properties of CAIX, the interaction of H-APBC on CAIX implied a decrease in TME acidosis, which mitigated tumor metastasis. Finally, H-APBC could produce a large amount of ROS under laser irradiation and induce irreversible tissue damage through thermal ablation, thus facilitating apoptosis of tumor cells. Taken together, H-APBC nanocomplexes might be an effective mTOR inhibition-based cancer treatment strategy that could resist both hypoxia adaptation and TME acidosis in solid tumors.

## Supplementary Information

**Additional file 1: Figure S1.** SEM images of nanoparticles. **Figure S2.** The release profiles of Ce6 (**a**) and BEZ235 (**b**) from H-APBC at pH 7.4, 6.5 and 5.6 PBS. **Figure S3.** Absorption spectrum and calibration curve of component. **a** Vis-NIR spectra of H-APBC NPs at various concentrations. **b** Calibration curve of H-APBC NPs at 808 nm. **Figure S4.** CLSM images of 4T1 cells after 6 h incubation with H-APBC under hypoxia. Scale bar: 25 μm. **Figure S5.** Images of migrated 4T1 cells incubated with different treatment under normoxia. Scale bar: 50 μm. **Figure S6.** Flow cytometry analysis of the proportions of 4T1 cells co-stained with Annexin V-FITC and PI in different treatments during the normoxic experiment. (n = 3). *p < 0.05, **p < 0.01, and ***p < 0.001. **Figure S7.** Viability of 4T1 cells in different groups under the normoxic condition. (n = 3). *p < 0.05, **p < 0.01, and ***p < 0.001. **Figure S8.** Immunofluorescence images of ROS detection in ABS, BEZ235, H-APBC and HT groups. Scale bar: 80 μm. **Figure S9.** Treatment effects of H-APBC. H&E (**a**), TUNEL (**b**), Ki67 (**c**) staining of tumor tissues in HD and HT groups. Scale bar: 80 μm. **Figure S10.** Images of expression on HIF-1α and CD31 in HD and HT groups. Scale bar: 80 μm (HIF-1α), 120 μm (CD31). **Figure S11.** Physiological function assessment of liver and kidney toxicity from the treated mice. **a**–**f** Total bilirubin (TBIL), direct bilirubin (DBIL), total bile acids (TBA), creatinine (CR), blood urea nitrogen (BUN) and uric acid (UA) levels recorded for mice in the treatment groups. The error bars are based on the SD of five mice. **Figure S12.** H&E staining of the main organs. Scale bar: 80 μm.

## Data Availability

The data are available in the main manuscript, Additional files, and from the corresponding authors upon reasonable request.
