# Supplementary material for: Carbonic anhydrase IX-targeted H-APBC nanosystem combined with phototherapy facilitates the efficacy of PI3K/mTOR inhibitor and resists HIF-1α-dependent tumor hypoxia adaptation
Source: J Nanobiotechnology. 2022 Apr 12;20:187. doi: 10.1186/s12951-022-01394-w (PMC9004111; doi:10.1186/s12951-022-01394-w)
Supplement: Supplementary file 1 — Additional file 1: Figure S1. SEM images of nanoparticles. Figure S2. The release profiles of Ce6 (a) and BEZ235 (b) from H-APBC at pH 7.4, 6.5 and 5.6 PBS. Figure S3. Absorption spectrum and calibration curve of component. a Vis-NIR spectra of H-APBC NPs at various concentrations. b Calibration curve of H-APBC NPs at 808 nm. Figure S4. CLSM images of 4T1 cells after 6 h incubation with H-APBC under hypoxia. Scale bar: 25 μm. Figure S5. Images of migrated 4T1 cells incubated with different treatment under normoxia. Scale bar: 50 μm. Figure S6. Flow cytometry analysis of the proportions of 4T1 cells co-stained with Annexin V-FITC and PI in different treatments during the normoxic experiment. (n = 3). *p < 0.05, **p < 0.01, and ***p < 0.001. Figure S7. Viability of 4T1 cells in different groups under the normoxic condition. (n = 3). *p < 0.05, **p < 0.01, and ***p < 0.001. Figure S8. Immunofluorescence images of ROS detection in ABS, BEZ235, H-APBC and HT groups. Scale bar: 80 μm. Figure S9. Treatment effects of H-APBC. H&E (a), TUNEL (b), Ki67 (c) staining of tumor tissues in HD and HT groups. Scale bar: 80 μm. Figure S10. Images of expression on HIF-1α and CD31 in HD and HT groups. Scale bar: 80 μm (HIF-1α), 120 μm (CD31). Figure S11. Physiological function assessment of liver and kidney toxicity from the treated mice. a–f Total bilirubin (TBIL), direct bilirubin (DBIL), total bile acids (TBA), creatinine (CR), blood urea nitrogen (BUN) and uric acid (UA) levels recorded for mice in the treatment groups. The error bars are based on the SD of five mice. Figure S12. H&E staining of the main organs. Scale bar: 80 μm. [file 12951_2022_1394_MOESM1_ESM.docx]

**Additional file 1**

**Carbonic anhydrase IX-targeted H-APBC nanosystem combined with phototherapy facilitates the efficacy of PI3K/mTOR inhibitor and resists HIF-1α-dependent tumor hypoxia adaptation**

*Jie Liu*^a†^*, Xiaochun Hu*^a†^*, Lei Feng*^a^*, Yun Lin*^a^*, Shujing Liang*^a^*, Zhounan Zhu*^a^*, Shuo Shi*^a,^ ** and Chunyan Dong*^a,^ ***

^a^Breast Cancer Center, Shanghai East Hospital, Shanghai Key Laboratory of Chemical Assessment and Sustainability, School of Chemical Science and Engineering, Tongji University, Shanghai, 200092, P. R. China.

E-mail address: cy_dong@tongji.edu.cn; [shishuo@tongji.edu.cn](mailto:shishuo@tongji.edu.cn)


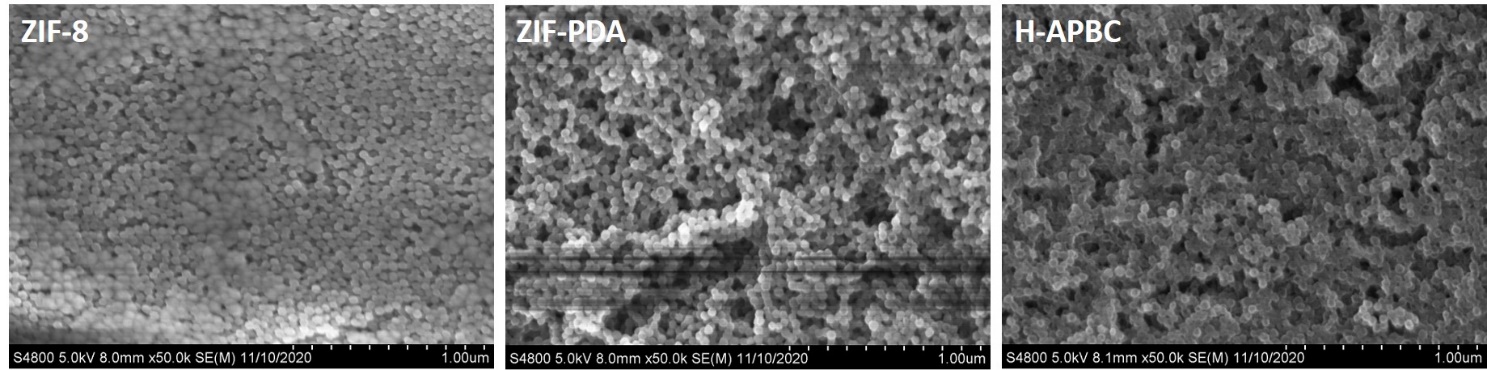


Fig. S1 SEM images of nanoparticles.


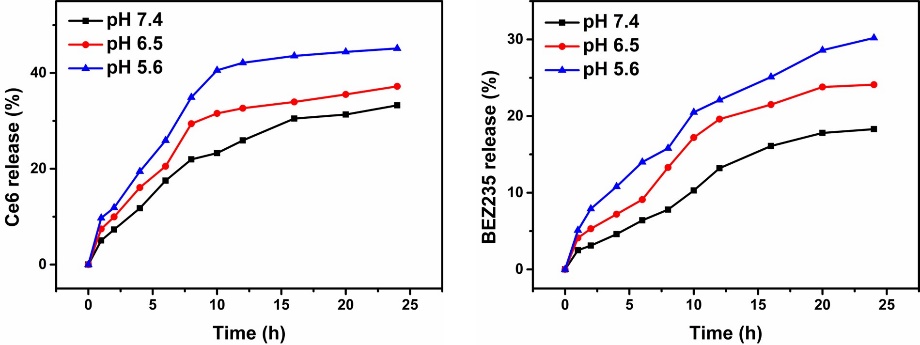


Fig. S2 The release profiles of Ce6 (a) and BEZ235 (b) from H-APBC at pH 7.4, 6.5 and 5.6 PBS.


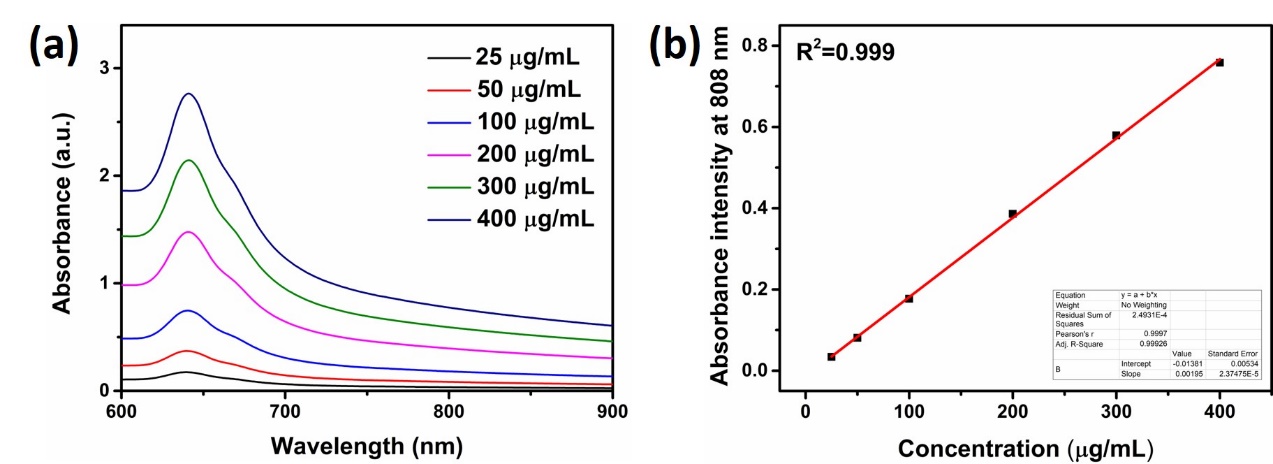


Fig. S3 Absorption spectrum and calibration curve of component. a) Vis-NIR spectra of H-APBC NPs at various concentrations. b) Calibration curve of H-APBC NPs at 808 nm.


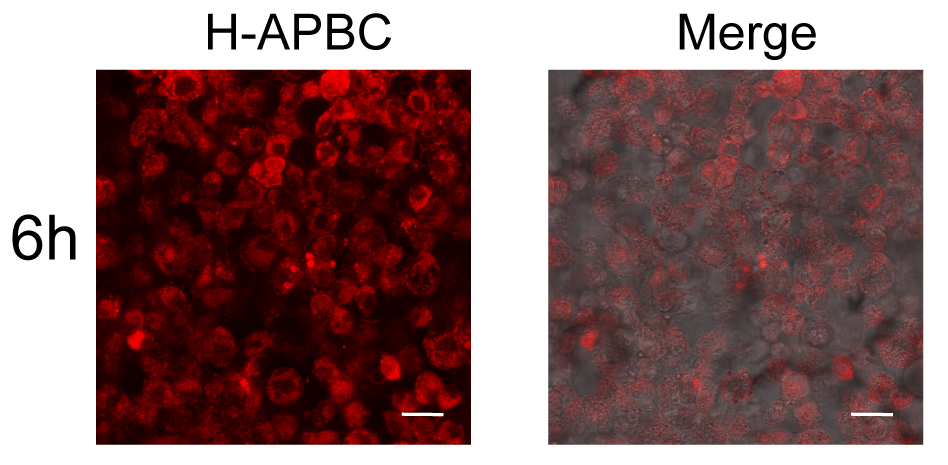


Fig. S4 CLSM images of 4T1 cells after 6h incubation with H-APBC under hypoxia. Scale bar: 25 μm.


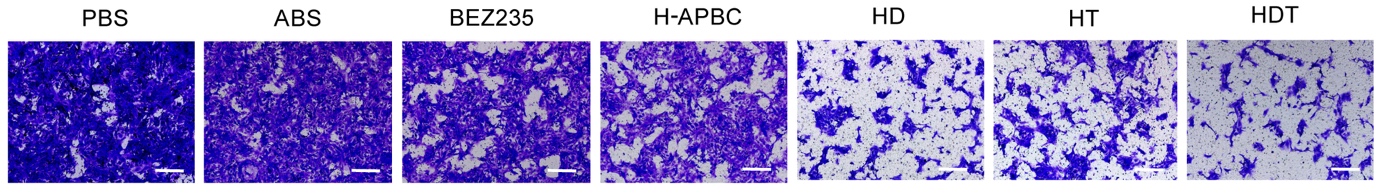


Fig. S5 Images of migrated 4T1 cells incubated with different treatment under normoxia. Scale bar: 50 μm.


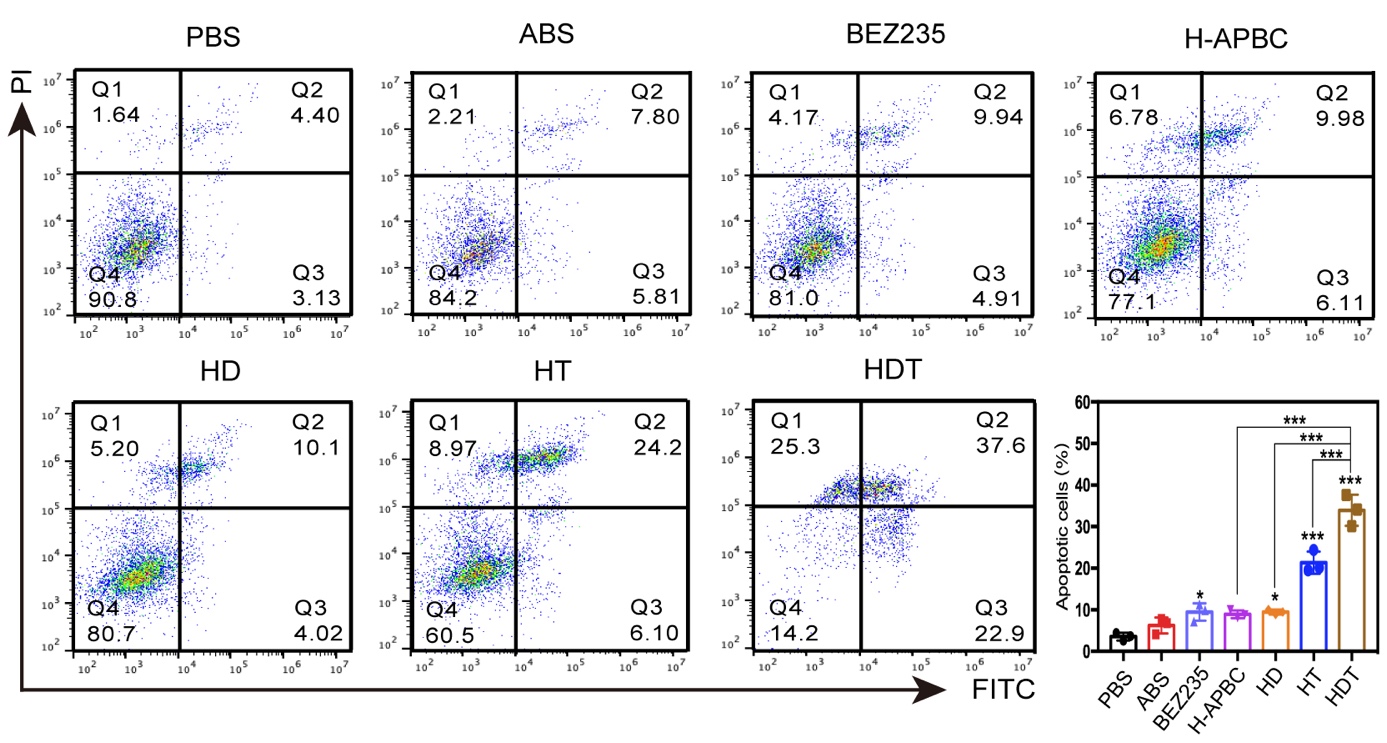


Fig. S6 Flow cytometry analysis of the proportions of 4T1 cells co-stained with Annexin V-FITC and PI in different treatments during the normoxic experiment. (n = 3). *p < 0.05, **p < 0.01, and ***p < 0.001.


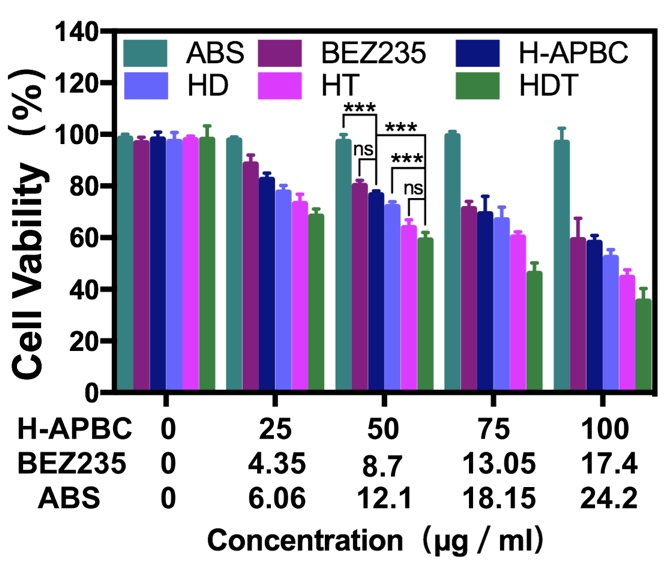


Fig. S7 Viability of 4T1 cells in different groups under the normoxic condition. (n = 3). *p < 0.05, **p < 0.01, and ***p < 0.001.


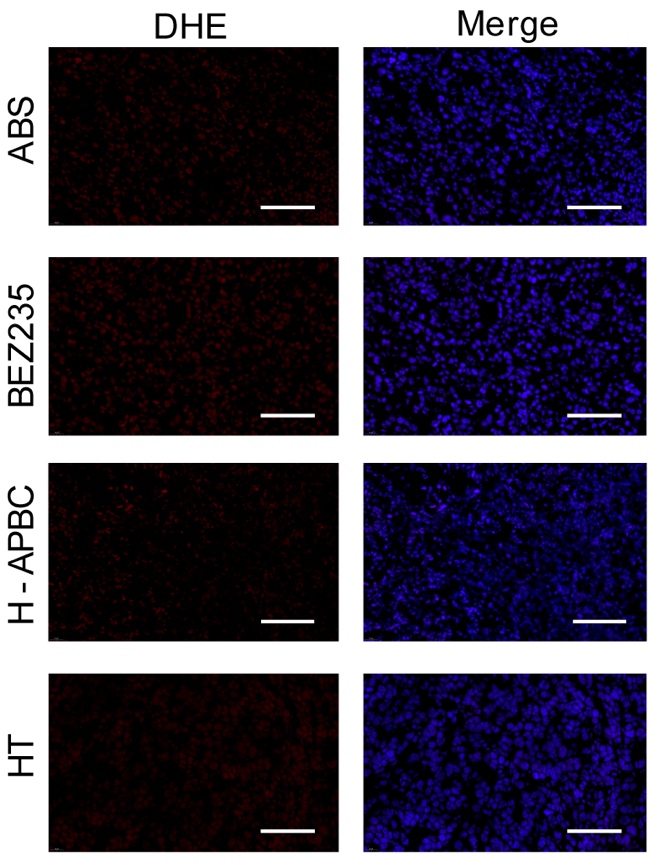


Fig. S8 Immunofluorescence images of ROS detection in ABS, BEZ235, H-APBC and HT groups. Scale bar: 80 μm


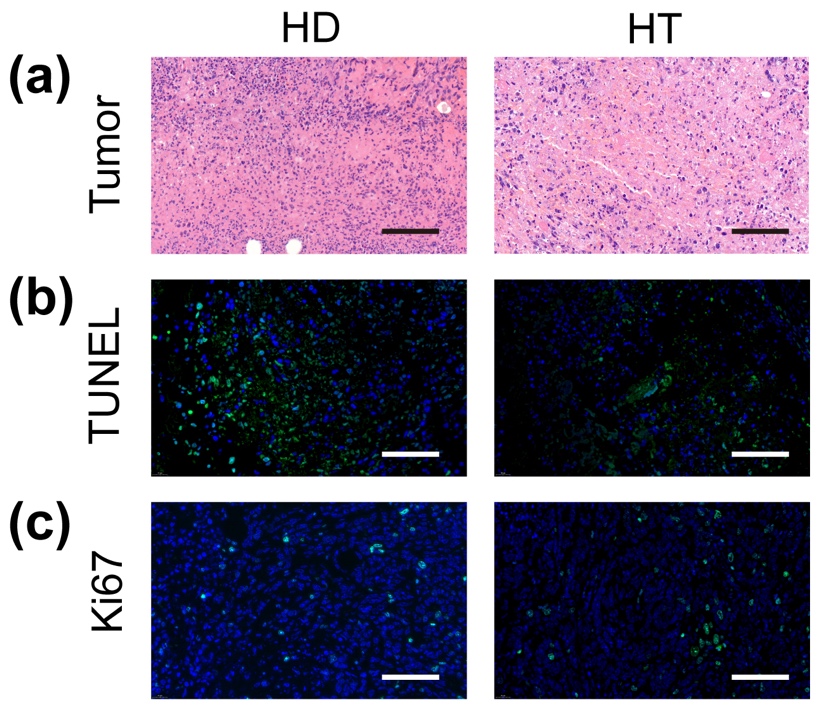


Fig. S9 Treatment effects of H-APBC. H&E a), TUNEL b), Ki67 c) staining of tumor tissues in HD and HT groups. Scale bar: 80 μm


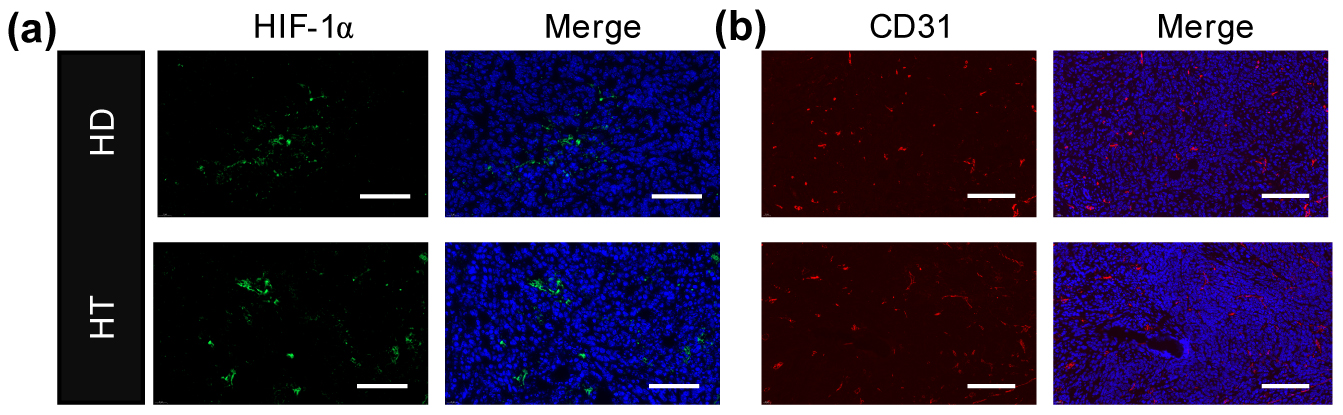


Fig. S10 Images of expression on HIF-1α and CD31 in HD and HT groups. Scale bar: 80 μm (HIF-1α), 120 μm (CD31).


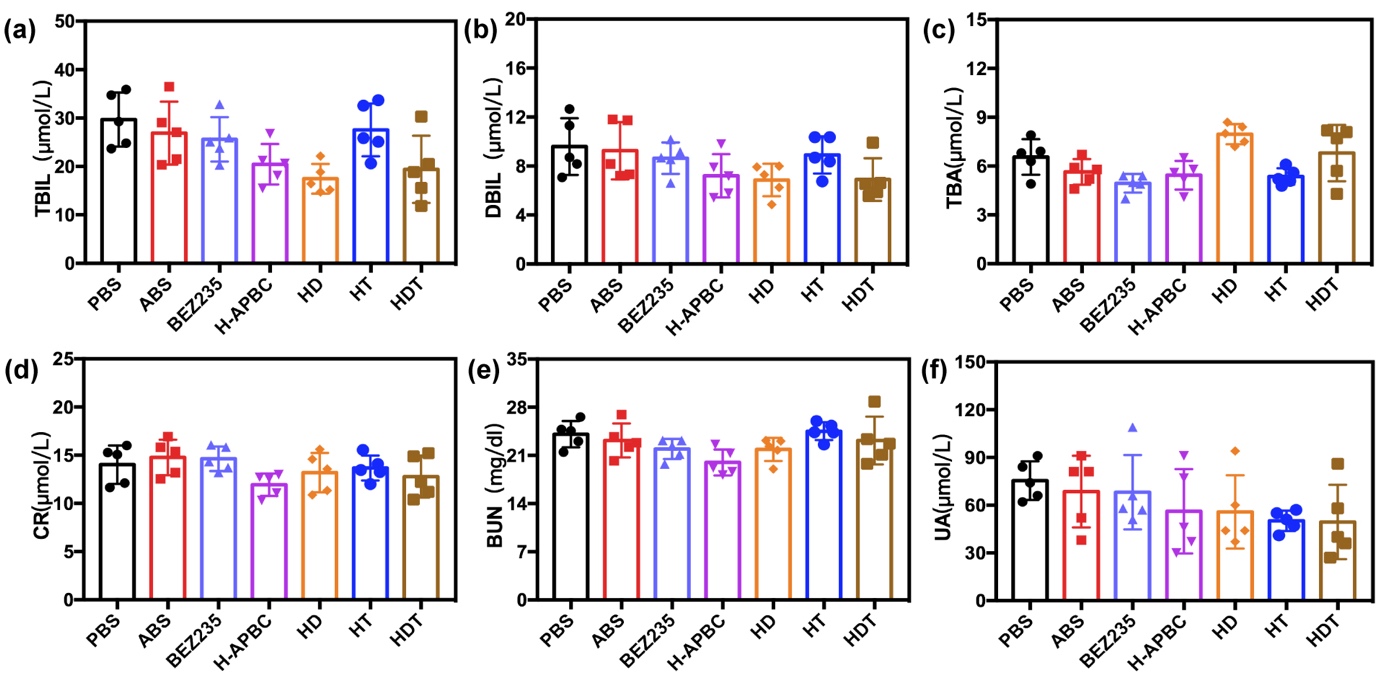


Fig. S11 Physiological function assessment of liver and kidney toxicity from the treated mice. a-f) Total bilirubin (TBIL), direct bilirubin (DBIL), total bile acids (TBA), creatinine (CR), blood urea nitrogen (BUN) and uric acid (UA) levels recorded for mice in the treatment groups. The error bars are based on the SD of five mice.


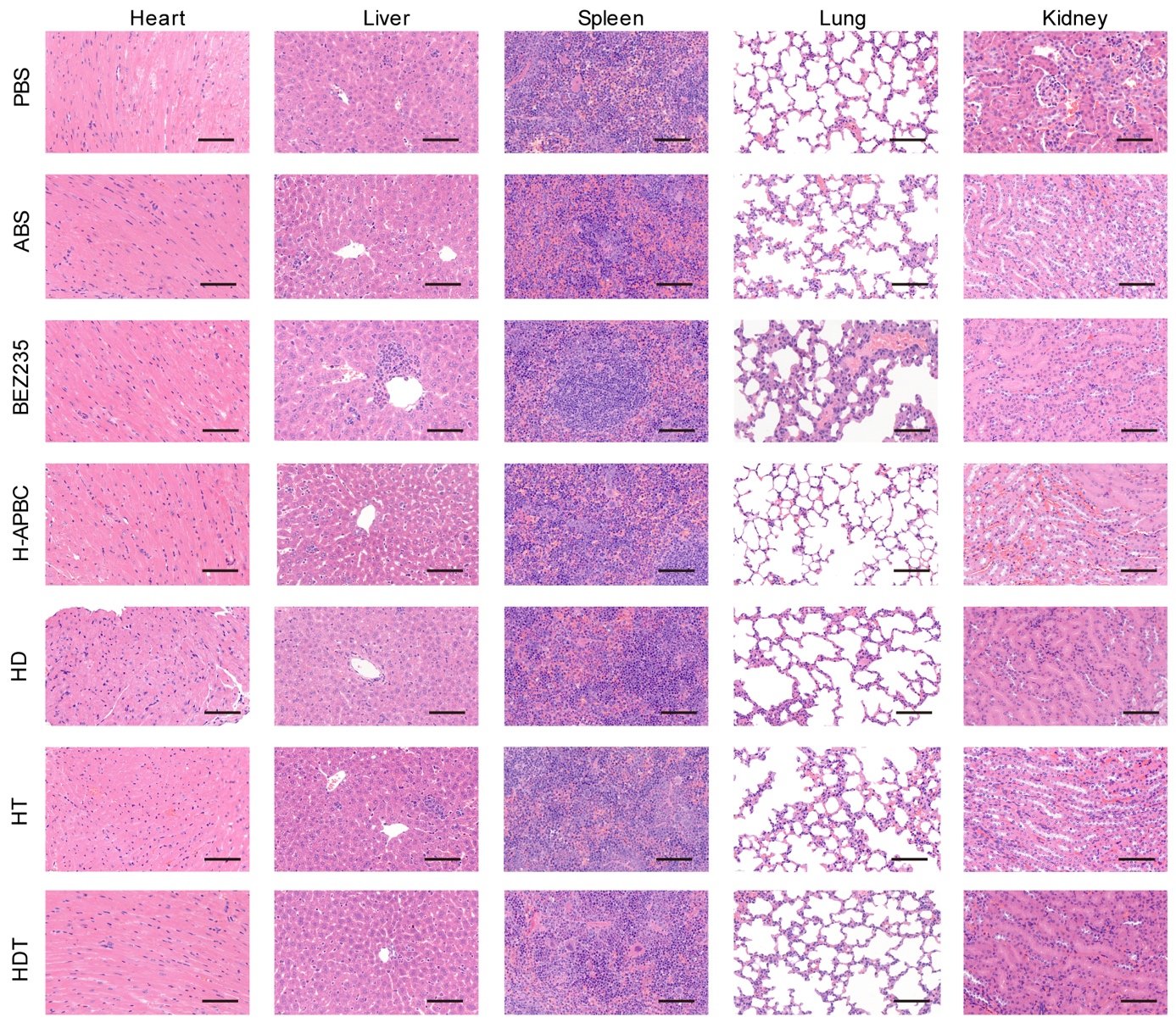


Fig. S12 H&E staining of the main organs. Scale bar: 80 μm
